# Prevalence and Predisposing Factors for Depressive Status in Chinese Patients with Obstructive Sleep Apnoea: A Large-Sample Survey

**DOI:** 10.1371/journal.pone.0149939

**Published:** 2016-03-02

**Authors:** Yaozhang Dai, Xuewu Li, Xin Zhang, Sihua Wang, Jianzhong Sang, Xiufen Tian, Hua Cao

**Affiliations:** 1 Department of Otolaryngology Head and Neck Surgery, the First Affiliated Hospital, Zhengzhou University, Zhengzhou, Henan, China; 2 Shenzhen Kangning Hospital, Shenzhen Key Lab for Psychological Healthcare, Shenzhen, Guangdong, China; 3 Department of Otolaryngology Head and Neck Surgery, Xiangya Hospital, Central South University, Changsha, Hunan, China; 4 School of Public Health, Zhengzhou University, Zhengzhou, Henan, China; Hospital General Dr. Manuel Gea González, MEXICO

## Abstract

**Background and Objective:**

Recently, there are few studies reporting on depressive status and obstructive sleep apnoea (OSA) in China. A large-sample survey was to be performed to explore the prevalence of depressive status and related factors in Chinese patients with OSA.

**Methods:**

From among a randomly-selected group of OSA patients, 1,327 met inclusion criteria. After screening with the Symptom Checklist 90 (SCL-90) and Self-Rating Depression Scale (SDS), patients were assigned to OSA without depressive status (control group, *n* = 698) and OSA with depressive status (*n* = 629) groups. Using chi-squared testing, the correlation analyses between the depressive status and OSA patient demographic and clinical variables were tested. Then depression-related risk factors in OSA patients were analysed using stepwise linear regression analysis. The effects of family and social factors on depressive status in OSA patients were investigated using Mann-Whitney U (one of nonparametric test).

**Results:**

The prevalence of depressive status was 47.4% in OSA patients. Depressive status was significantly associated with female gender, single status, Family Burden Scale of Disease (FBS), Family APGAR Index (APGAR), apnoea-hypopnea index (AHI), and Perceived Social Support Scale (PSSS). Stepwise linear regression analysis further indicated that single status, hypoxemia, APGAR, AHI, PSSS, AHI, and FBS were all risk factors for depressive status in OSA patients. The total of the FBS score and three of its sub-factors scores (family daily activities, family relationships and mental health of family members) were higher, and the total of the APGAR score and two of its sub-factors scores (adaptability and affection) were lower in OSA with depressive status compared with the control group. Besides, the total score for the PSSS and scores for its two sub-factors (family support and social support) were all lower in OSA patients with depressive status than those of the control group.

**Conclusions:**

Depressive status has high comorbid rate in Chinese OSA patients and is significantly associated with single status, apnoea-hypopnea index, hypoxemia, family and social supports.

## Introduction

Obstructive sleep apnoea (OSA) is a sleep-related breathing disorder that is characterised by repeated episodes of upper airway occlusion during sleep, as well as symptoms such as sleepiness and snoring. In OSA, sleep often becomes fragmented due to repeated respiratory events, which are identified as apnea when airflow is stopped for ten or more seconds, or hypopnea when airflow decreases by 30% or more and is accompanied by a decrease in oxyhemoglobin saturation [1]. OSA has two significant characteristics: repeated sleep apnoea and intermittent hypoxemia [2]. The diagnosis of OSA is based on respiratory event index, apnoea-hypopnea index (AHI), or respiratory disturbance index (RDI) [3]. OSA increases the risk of poor neurocognitive function and organ dysfunction and increases the mortality of patients with hypertension and cardiovascular disease [4–6]. Furthermore, OSA patients show a high prevalence of depression, with up to half experiencing elevated depressive symptoms [7]. The depression symptoms in OSA patients reduce quality of life and compliance with continuous positive airway pressure treatment [8], and were correlated with progression of cardiovascular disease [9–12]. Unfortunately, in recent years, there are few studies reporting on depression and OSA in China. The objectives of this study were to analyse the data of 1,327 Chinese OSA patients, to explore the prevalence of depressive status, and to further investigate related factors that might be modified to decrease the comorbid rate of depressive status in OSA patients.

## Data and Methods

### Participants

Between October 2012 and February 2014, 1,513 participants were randomly selected among 2,464 patients who had been diagnosed for OSA by patient’s identification number (patients ID number) by computer at the First Affiliated Hospital of Zhengzhou University of China. All the 1,513 participants had been received polysomnography (PSG) at night for 7 hours. All data were analysed by a sleep technologist and then verified by a sleep physician. After application of inclusion and exclusion criteria, a total of 1,327 OSA patients and 1,327 their close contacts completed the study.

Inclusion criteria were: (i) satisfied the OSA diagnosis [13], and did not receive any treatment for OSA before completing the questionnaires assessment; (ii) aged 18 years or older, having normal communication ability, without mental retardation; (iii) signed informed consent; (iv) close contacts in the family of the OSA patient, without mental or neurological disorders, who could normally communicate.

Exclusion criteria were: (i) previous diagnosis of mental illness except for depression; (ii) central nervous system injury or a history of central nervous system injury which could involve respiratory centres in the brain; (iii) other severe physical illness, *e*.*g*., tumour, hemiplegia, which could increase the risk of associated depressive symptoms; (iv) reliance on anti-psychotic, antidepressant or anti-anxiety drugs, alcohol, or psychoactive substances; (v) mental disorder in any lineal relatives; (vi) survey data that were not detailed or were unreliable.

### Definition of variables

Demographic and clinical variables were completed by a general questionnaire. Demographic variables included age, gender, marital status (married, single if unmarried, divorced and widowed), job category, smoking status, alcohol intake, education level (lower if ≤Primary school, Secondary if receive junior and or senior middle school, higher education if ≥College).

According to previous literatures [14, 15], information about smoking and alcohol consumption was collected. Specifically, non-smokers were those who had not smoked during the last month and had never smoked for longer than a year. Subjects were classified as a former smoker when they reported that they had smoked during a whole year, had not smoked during the last month and stopped smoking. Those who had smoked for longer than a year and had not stopped smoking were classified as current smoker. Total tobacco use of the current smokers was estimated by using the following quantities: 1 cigarette = 1 g tobacco, 1 cigarillo = 3 g tobacco and 1 cigar = 5 g tobacco. Moderate smoking was defined as 20 g/day or less, and heavy as more than 20 g/day.

Alcohol intake was based on the response to specific questions regarding intake frequency and the average number of units consumed on a drinking day. Individuals who reported not having consumed alcohol during the past month were considered non-drinkers. The number of alcoholic drinks per week was determined by multiplying the number of drinking days per week by the average number of units consumed on a drinking day. We then divided the number of alcoholic drinks/week by 7 in order to arrive at the number of alcoholic drinks per day. Individuals were classified into four groups according to their daily alcohol intake: 0 drinks/day (non-drinker),≤1 drink/day (light drinker), >1 to 2 drinks/day (moderate drinker) and >2 drinks/day (heavy drinker).

Clinical variables were collected by investigators according to the medical records such as PSG, diagnosis time, disease type, and so on. The AHI (mean number of apneas + hypopneas per hour of sleep) and hypoxemia (a decrease in oxyhemoglobin saturation during apneas and hypopneas) are both the most often-reported statistically significant predictor of OSA. An AHI of 5–15 is considered as mild, 16–30 as moderate, and > 30 as severe OSA [12, 13]. Hypoxemia is classified as mild (SaO_2_ (%) 85–92), moderate (SaO_2_ (%) 65–84) or severe (SaO_2_ (%) < 64) [13]. body mass index (BMI) classes: normal weight (BMI<25.0 kg/m^2^), overweight (BMI 25.0 to 35.0 kg/m^2^) or obese (BMI≥≥35.0 kg/m^2^). Heart diseases included coronary heart disease, arrhythmias and heart failure. Diabetes Mellitus contains Type I and Type II diabetes mellitus.

### Research tools

The following questionnaires were used in this study: the Symptom Checklist 90 (SCL-90), the Self-rating Depression Scale (SDS), the Family Burden Scale of Disease (FBS), the Family APGAR Index (APGAR) and the Perceived Social Support Scale (PSSS). Upon testing, these questionnaires showed good reliability and validity in China [16–21]. (i) Symptom Checklist 90 (SCL-90): The SCL-90, designed by Derogatis in 1975, is a self-administered 90-item questionnaire with a five-point Likert scale (e.g., 1, not at all; 5, extremely often), which evaluates a broad range of symptoms of psychopathology [22]. It measures nine symptom dimensions including somatization, interpersonal sensitivity, obsessive-compulsive symptoms, depression, anxiety, hostility, phobic anxiety, paranoid ideation, and psychoticism, as well as a class of additional items that assess other aspects of symptoms. Anxiety symptoms appear in some depressed patients, so patients scoring > 160 on the SCL-90 or having scores ≥ 3.0 for the depression or the anxiety items were asked to complete the Self-rating Depression Scale (SDS) survey. (ii) The SDS, designed by Zung in 1965, is generally considered a reliable instrument for measuring depressive symptoms in primary care [23]. It is a self-reported 20-item questionnaire. Item responses are rated from 1 to 4, with higher scores corresponding to more frequent symptoms. The standardized score (the total of the raw item scores of the 20 items multiplied by 1.25) represents the severity of depressive symptoms. The clinical threshold is 53; scores in the 53–62, 63–72 and 72–100 ranges correspond to mild, moderate, and severe depression, respectively [24]. (iii)Family Burden Scale of Disease (FBS): The FBS, originally designed by Pai and Kapur, is used to measure the burden of disease on the family of a patient [25]. It is a 24-item questionnaire, containing six factors: family economic burden (factor 1, 6 items), family daily activities (factor 2, 5 items), family leisure and recreational activities (factor 3, 4 items), family relationships (factor 4, 5 items), physical health of family members (factor 5, 2 items) and mental health of family members (factor 6, 2 items). The number of items of each factor is different. For comparison, according to Lv F’s revision[26], the score for each factor and total score for six factors were separately divided by the number of items, then the standardized average score for each factor and the total average score were obtained. The average score is considered as a boundary Three levels were re-assigned (if scoring 0, assignment = 0; if < average score, assignment = 1; if ≥ average score, assignment = 2), specifically, no burden, scoring 0; moderate burden, scoring 1; severe burden, scoring 2. (iv)Family APGAR Index (APGAR): The Family APGAR Index, designed by Smilkstein in 1978, is introduced to assess adult satisfaction with social support received within a family [27]. It consists of statements on five parameters of family functioning: Adaptability, Partnership, Growth, Affection and Resolve. The family member’s response is based on the frequency of feeling satisfied with each of the five parameters using a 3-point scale ranging from 0 (hardly ever) to 2 (almost always). The total score ranges from 0 to 10, with a higher total score indicating better family functioning. A score of 0–3 denotes a severely dysfunctional family, 4–7 a moderately dysfunctional family and 8–10 a highly functional family. (v) Perceived Social Support Scale (PSSS): The PSSS, introduced by Blumenthal and designed by Zimet, is a 12-item self-reported assessment of perceived emotional support from friends, family and significant others [28]. According to the revisions by Jiang [29], the 12 items are divided into “family support” and “social support” categories. The former is the sum of scores from items 3, 4, 8 and 11, and the latter is the sum of scores from the other items. Responders indicate their degree of agreement with the items on a 7-point Likert-type scale ranging from “very strongly disagree” to “very strongly agree.” Items are summed to obtain scores between 12 and 84, with higher scores indicating greater support. A score less than 32 denotes a severely dysfunctional Social support system, and more than 50 denotes a well-functioning one.

### Survey methodology

This study was approved by the Ethics Committee of the First Affiliated Hospital of Zhengzhou University of China and written informed consent was obtained. Before the self-assessment, the well trained investigators should ensure the respondents clearly understand the filling method and the meaning of each question then make an independent self-assessment which should not affected by anyone. For the respondents with lower education(n = 36, 0.3%), the investigators read the scale item by item for them to make sure that all items in the scales were properly understood and the respondents make their own judgment. Investigators filled in the general questionnaire for the OSA patients and their close contacts (spouse, cohabiting peer, child or nursing workers). All patients separately filled in the SCL-90, the APGAR and the PSSS questionnaires. Patients scoring > 160 on the SCL-90 or having scores ≥ 3.0 for the depression or the anxiety items were asked to complete the SDS survey as well. It should be indicated that this study used two questionnaires (SCL-90 and SDS) rather than diagnostic interviews based on DSM-IV-TR to diagnose depressive disorder(s). Therefore, “depression” in this survey more referred to depressive status rather than depressive disorders. Designated by the patient, the close contacts thought to have a full understanding of the condition of the patient filled in the FBS questionnaires when not in the presence of the patients.

### Statistical treatment

The demographic and clinical variables of OSA patient are categorical variables, the correlation analyses between the depressive status and OSA patient characteristics were tested by a χ^2^ test. Depressive status-related risk factors in OSA patients were analysed using stepwise linear regression analysis. Whether comorbid depressive status is the dependent variable, and the independent variables which was significantly associated with the high prevalence of depressive status in OSA patients in the χ2 test will be introduced in stepwise linear regression equation. In order to assure the significance of independent variables in the equation, when a new variable is introduced into the equation, an F-test based on partial regression equations is performed to test the significance of every independent variable in the equation, degenerated variables will be removed as they no longer have significant influence. The two-way screening is repeated until no variable need to be introduced or removed, in which case the regression equation is locally optimal. The differences in family burden, family care and emotional support between patients with depressive status vs. without were all tested by Mann-Whitney U test (the one of nonparametric test). Statistical analysis was performed using the SPSS software (version 17.0), and *P* <0.05 was considered statistically significant.

## Results

### General condition and depressive status in 1,327 OSA patients

A total of 1,327 OSA patients met the requirements and completed the whole study. As shown in Table 1, there were 1,071 male and 256 female OSA patients, with a median age of 47 years (range, 18–82). Among the patients, 1,115 were married, while 212 patients were single (110 patients unmarried, 27 patients divorced, and 75 patients widowed). There were 525 patients who engaged mainly in physical work, and the remainder engaged in mainly non-physical work. Among the study respondents, 200(15.1%) and 504(37.9%) patients reported that they were moderate and heavy smokers respectively. The alcohol consumption groups (light, moderate and heavy drinker) are 190(14.3%), 267(20.1%) and 160(12.1%) respectively. 1155(87.0%) patients received secondary education, while 36(2.7%) patients only experienced a lower education. Among these OSA patients, 903 (68%), 401 (30.2%), and 358 (26.98%) individuals had comorbid hypertension, heart disease and diabetes mellitus respectively.

**Table 1:** Association between demographic characteristics and depressive status of the OSA patients.

Characteristics	OSA without depressive status, n(%)	OSA with depressive status, n(%)	*P-*Value
**Age**			
≤47	373 (28.1%)	319 (24.0%)	0.778
>47	325 (24.5%)	310 (23.4%)	
**Gender**			
Male	587 (44.2%)	484 (36.5%)	0.001
Female	111 (8.4%)	145 (10.9%)	
**Marital status**			
Single	84 (6.3%)	128 (9.6%)	0.000
Married	614 (46.3%)	501 (37.8%)	
**Job category**			
Physical work	258 (19.4%)	267 (20.1%)	0.059
Mental work	440 (33.2%)	362 (27.3%)	
**Education level**			
≤Primary school	20 (1.5%)	16 (1.2%)	0.052
Junior middle school	462 (34.8%)	373 (28.1%)	
Senior middle school	150 (11.3%)	170 (12.8%)	
≥College	66 (4.9%)	70 (5.3%)	
**Smoking status**			
Non-smoker	264 (19.9%)	250 (18.8%)	0.176
Former smoker	68 (5.1%)	41 (3.1%)	
<20 gram tobacco/day	108 (8.1%)	92 (6.9%)	
≥20 gram tobacco/day	258 (19.4%)	246 (18.5%)	
**Alcohol intake**			
Non drinker	390 (29.4%)	320 (24.1%)	0.059
≤1 drink/day	100 (7.5%)	90 (6.8%)	
>1 to 2 drinks/day	121 (9.1%)	146 (11.0%)	
>2 drinks/day	87 (6.6%)	73 (5.5%)	
**BMI(kg/ m**^**2**^**)**			
<25	215 (16.2%)	212 (16.0%)	0.789
25–35	232 (17.5%)	207 (15.5%)	
>35	251 (18.9%)	201 (15.1%)	
**AHI**			
5–20	221 (16.7%)	174 (13.1%)	0.036
21–40	240 (18.1%)	207 (15.6%)	
>40	237 (17.9%)	248 (18.7%)	
**Hypoxemia(%)**			
< 65	239 (18.0%)	166 (12.5%)	0.001
65–84	229 (17.3%)	201 (15.1%)	
85–92	230 (17.3%)	262 (19.7%)	
**FBS**			
0	246 (18.5%)	251 (18.9%)	0.007
1	205 (15.4%)	173 (13.0%)	
2	247 (18.6%)	205 (15.4%)	
**APGAR**			
0–3	202 (15.2%)	224 (16.9%)	0.022
4–6	232 (17.5%)	207 (15.6%)	
7–10	264 (19.9%)	198 (14.9%)	
**PSSS**			
< 32	244 (18.4%)	251 (18.9%)	0.030
33–50	220 (16.6%)	211 (15.9%)	
>50	234 (17.6%)	167 (12.6%)	
**Concomitant disease**			
**Hypertension**			
Yes	457 (34.4%)	446 (33.6%)	0.113
No	241 (18.2%)	183 (13.8%)	
**Heart diseases**			
Yes	228 (17.2%)	173 (13.0%)	0.115
No	470 (35.4%)	456 (34.4%)	
**Diabetes Mellitus**			
Yes	198 (14.9%)	160 (12.1%)	0.385
No	500 (37.7%)	469 (35.3%)	

**Abbreviations:** SDS, Self-rating Depression Scale; BMI, body mass index; FBS, Family Burden Scale of Disease; APGAR, Family APGAR Index; PSSS, Perceived Social Support Scale; AHI, apnoea-hypopnea index.

Of the 1,327 OSA patients, 629 (47.4%) patients had depressive status and the remainder were regarded as the control group. According to the SDS questionnaire, 380 (28.6%), 125 (9.4%), and 124 (9.3%) patients were classified as having mild, moderate and severe depressive status respectively. The percentage of female OSA patients among the total sample was low (19.3%), but the prevalence of depressive status was relatively high among women (145/256, 56.6%). In married OSA patients, the prevalence of depressive status was 44.9%. Among singles, it reached up to 60.4%. The differences between OSA patients with and without depressive status were investigated by the χ^2^ test. The high prevalence of depressive status in OSA patients was significantly associated with single status (*p* = 0.000), female gender (*p* = 0.001), hypoxemia (*p* = 0.001), FBS (*p* = 0.007), APGAR (*p* = 0.022), PSSS (*p* = 0.030) and AHI (*p* = 0.036) (Table 1).

### Regression analysis of depressive status-related risk factors in OSA patients

Stepwise linear regression analysis was used to further screen risk factors for depressive status in OSA patients, the independent variables (gender, marital status, FBS, APGAR, AHI, PSSS and hypoxemia) significantly associated with the high prevalence of depressive status in OSA patients in the χ2 test were introduced in their categorical form similar to that in Table 1. Result of stepwise regression equation variance analysis is F = 8.645, *p* = 0.000, indicated that stepwise fitted multiple linear regression equation is statistically significant. Results revealed that single marital status, hypoxemia, APGAR, PSSS, AHI, FBS were all risk factors for the high comorbidity of depressive status in OSA patients, and single status and hypoxemia are the most influential factors to high prevalence of depressive status in OSA patients (Table 2).

**Table 2:** Stepwise linear regression analysis of depressive status-related risk factors in OSA Patients.

	B	SE	t	*P*
**(Constant)**	1.352	0.084	16.091	0.000
**Marital status**	0.135	0.037	3.638	0.000
**Hypoxemia**	0.055	0.017	3.297	0.001
**APGAR**	-0.051	0.017	-3.061	0.002
**PSSS**	-0.045	0.017	-2.704	0.007
**AHI**	0.036	0.017	2.176	0.030
**FBS**	-0.034	0.016	-2.135	0.033

**Abbreviations:** B, regression coefficient; SE, Standard Error; APGAR, Family APGAR Index; PSSS, Perceived Social Support Scale; AHI, apnoea-hypopnea index; FBS, Family Burden Scale of Disease.

### Family and social factors for the prevalence of depressive status in OSA patients

To further explore the influence of the relevant family and social factors for the prevalence of depressive status in OSA patients, detailed analyses were performed APGAR, and PSSS. As displayed in Table 3, significant differences in total APGAR scores and sub-scores for factor 1 (adaptability) and factor 4 (affection) were visible between patients with depressive status vs. without (*p* < 0.05). The total score for the APGAR and the two scores for the abovementioned factors were all lower in OSA patients with depressive status than those of without. As shown in Table 4, significant differences in total score for the PSSS and scores for its two sub-factors (family support and social support) were all observed between patients with depressive status vs. without (*p* < 0.05). Moreover, the above PSSS scores were all lower in OSA patients with depressive status than those of the control group.

**Table 3:** Mann-Whitney U test of family care of OSA patients with depressive status by Family APGAR scale.

Category	n	APGAR	Factor 1	Factor 2	Factor 3	Factor 4	Factor 5
**OSA with Depressive status**	629	633.12*	625.62*	654.36*	660.74*	609.89*	664.03*
**Controls**	698	691.82*	698.59*	672.68*	666.94*	712.77*	663.97*
**Z**		-2.956	-3.752	-0.933	-0.313	-5.211	-0.003
***P***		0.003	0.000	0.351	0.754	0.000	0.998

*Rank mean; APGAR, Family APGAR Index

**Table 4:** Mann-Whitney U test of emotional support for OSA patients with depressive status by Perceived Social Support Scale.

Category	n	PSSS	Factor 1	Factor 2
**OSA with depressive status**	629	636.69*	625.46 *	641.13*
**Controls**	698	688.61*	698.73*	684.61*
**Z**		-2.618	-3.509	-2.076
***P***		0.009	0.000	0.039

* Rank mean; PSSS, Perceived Social Support Scale

### The family burden of the depressive status in OSA patients

As exhibited in Table 5, significant differences in total score for the FBS and three of its sub-factors (family daily activities, family relationships and mental health of family members) were all detectable between patients with depressive status vs. without (*p* < 0.05). Importantly, the total score for the FBS and three of its factors (family daily activities, family relationships, and mental health of family members) were all higher in OSA patients with depressive status than those of without.

**Table 5:** Mann-Whitney U test of family burden of OSA patients with depressive status by Family Burden Scale.

Category	n	FBS	Factor 1	Factor 2	Factor 3	Factor 4	Factor 5	Factor 6
**OSA with depressive status**	629	647.17*	646.95*	698.34*	648.18*	685.12*	648.09*	684.16*
**Controls**	698	679.16*	679.37*	633.05*	678.25*	644.97*	678.33*	645.84*
**Z**		-1.614	-1.737	-3.629	-1.600	-2.113	-1.589	-2.026
***P***		0.106	0.082	0.000	0.110	0.035	0.112	0.043

* Rank mean; FBS, Family Burden Scale of Disease

## Discussion

The prevalence rate of depression in OSA patient was 5%–63%[30], higher compared with patients without OSA that was generally 1.8%–3.3%[31]. Previous studies in China also found the similar results. The prevalence of depression was about 2.8%–4.4% in China [32], while it was 42.0%–44.19% in OSA population [33, 34]. In the present study, 47.4% Chinese OSA patients suffered from depressive status. The mechanism of the high prevalence of depressive status in OSA patients remains to be determined. Disordered sleep structure and hypoxemia during sleeping impact the emotions of OSA patients. The complaints from people around on frequent and loud snoring made by OSA patients may increase their low self-esteem and depressive status. In addition, excessive daytime sleepiness, reduced quality of life, and chronic complications (e.g., hypertension, heart disease, or diabetes mellitus) further aggravate their depressive symptoms [6, 35]. The results of this study may support the partial hypotheses abovementioned. In this study, AHI and hypoxemia are associated with the prevalence of depressive status, and are both risk factors for OSA patients with depressive status. In this survey, the proportion of female OSA patients was only 19.3%, but the prevalence rate of depressive status in women was higher than in men (56.6% vs 48.4%; *p* < 0.05). This finding suggests that although OSA was detected mainly in men, depressive status may be more common in female OSA patients [36, 37]. However, regression analysis demonstrated that gender was not a risk factor for the prevalence of depressive status in OSA patients. Besides, the prevalence rate of depressive status in married OSA patients was 44.9%, lower than 60.4% in singles (unmarried, divorced or widowed). Besides, stepwise linear regression analysis showed that single status is a most influential factors to high prevalence of depressive status in OSA patients. It suggests that the strong support of the partner-retention (the important part of the family supports) may help reduce the prevalence of the depressive status in OSA patients.

Social support is considered an important intermediary factor for maintaining good psychological stress and health, reflecting mainly spiritual and material help and support given to individuals from the immediate family, relatives, friends, colleagues, and social groups. According to previous studies, a correlation exists between the social support and depression, that is, a good social support can reduce the prevalence of depressive status [38]. Wang et al. found that a comprehensive treatment on OSA patients with depressive status, including the social support, has a significant improvement effect on their psychological status [39]. The marital status can reflect the family support level of a subject in a general extent, psychological and social factors such as inharmonious marriage however are one of the important pathogenic factors of depression and can then affect the prognosis [40]. Therefore, we further studied the impact of the family concern that OSA patients actually receive on the prevalence of depressive status. This study confirmed that significant differences in total score for the APGAR and two of its factors (adaptability and affection) were detectable between OSA patients and the control group. Stepwise linear regression analysis demonstrated that APGAR was a risk factor for the presence of depressive status in OSA patients. It suggested that reduced care and support from family or decreased sensitivity to these may be associated with high prevalence of depressive status in patients with OSA. This is because irritable negative emotional reactions generated in OSA patients after illness can be regulated with the family support system to reduce the development or aggravation of depressive status to some extent. In addition, the present study used PSSS to investigate the OSA patient’s subjective feelings about social support, and demonstrated that total scores for PSSS and two of its factors (family support and social support) were all lower in OSA patients with depressive status compared with the control group. These data suggest that reduced care and support from family and community, or decreased sensitivity to care, respect, and support may be associated with high prevalence of depressive status in patients with OSA. Therefore, more care and support should be provided to OSA patients by the patient’s family, as well as society, to help reduce the prevalence of depressive status.

On the other side, the same statistically significant differences in total scores for the FBS and three of its factor scores (family daily activities, family relationships, and mental health of family members) were visible. This indicated that the combination of depressive status with OSA not only greatly affects the patients themselves, but also impacts their family members. As a result, care and support for OSA patients with depressive status could be further reduced in a family. It suggests that more care and supports should be paid to OSA patients and their family members (especially the partner of OSA patient) in order to reduce the prevalence of depressive status.

In summary, our results indicate the possibility of a high prevalence of depressive status in Chinese OSA patients. The prevalence of depressive status was strongly associated with single marital status, a high AHI score, hypoxemia, a lack of family and social supports. Thus, more attention should be paid to the risk of depressive status in OSA patients, and more care, respect, support, and help should be provided to OSA patients and their families to reduce predisposing factors for depressive status.

## Supporting Information

S1 Dataset The dataset of demographic characteristics and depressive status of the 1,327OSA patients. (XLS)
